# Pharmacogenetics of the Treatment of Neglected Diseases

**DOI:** 10.3390/genes16010054

**Published:** 2025-01-05

**Authors:** Tiffany Borges Cabral, Amanda Carvalho de Oliveira, Gisely Cardoso de Melo, Fernanda Rodrigues-Soares

**Affiliations:** 1 Department of Pathology, Genetics and Evolution, Federal University of Triângulo Mineiro, Uberaba 38025-180, Brazil; tiffanyborgescabral@gmail.com; 2 Postgraduate Program in Tropical Medicine, Amazonas State University (PPGMT/UEA), Manaus 69040-000, Brazil; amandacarvalho42@gmail.com (A.C.d.O.); cardosogisely@gmail.com (G.C.d.M.); 3 Tropical Medicine Foundation (FMT-HVD) Dr. Heitor Vieira Dourado, Manaus 69040-000, Brazil

**Keywords:** pharmacogenetics, treatment, HIV, tuberculosis, malaria

## Abstract

Background/Objectives: Pharmacogenetics (PGx) aims to identify individuals more likely to suffer from adverse reactions or therapeutic failure in drug treatments. However, despite most of the evidence in this area being from European populations, some diseases have also been neglected, such as HIV infection, malaria, and tuberculosis. With this review, we aim to emphasize which pharmacogenetic tests are ready to be implemented in treating neglected diseases that have some evidence and call attention to what is missing for these three diseases. Methods: A critical literature review on the PGx of HIV infection, malaria, and tuberculosis was performed. Results: There are three PGx guidelines for antiretroviral drugs used in HIV infection, one for malaria, and none for tuberculosis. Some evidence is already available, and some genes have already been identified, such as *CYP2D6* for primaquine treatment and *NAT2* for isoniazid. However, some barriers to the implementation are the lack of evidence due to the few studies on the diseases themselves and the admixture of the most affected populations, which must be considered, given the genetic differentiation of these populations. Conclusions: PGx tests such as abacavir are already implemented in some places, and efavirenz/atazanavir is ready to implement if this medication is used. Other gene–drug associations were found but still do not present a clear recommendation. We call attention to the need to generate more evidence for testing treatments for other neglected diseases, such as malaria and tuberculosis, given their epidemiological importance and for the public health of less favored populations.

## 1. Introduction

In 1970, the term “neglected diseases” was used for the first time to include diseases caused by bacterial infections, helminths, parasites, and viruses. They occur mainly in less favored populations, with little access to health, education, basic sanitation, housing, and other factors, being a significant challenge for public health. These diseases include, among others, Chagas, schistosomiasis, leishmaniasis, dengue, malaria, HIV/AIDS, and tuberculosis [1].

The treatment for these diseases is also a challenge because these populations have low access to medication, and treatments often include drugs with narrow therapeutic indexes and a high probability of causing adverse reactions or therapeutic failure in some individuals.

When a patient undergoes drug treatment, it is expected that the target will be reached and the therapy will be successful, regardless of the pathology being treated. The doses required to maintain the plasma concentration of a drug within the therapeutic window may vary due to several factors, such as age, sex, ethnicity, diseases, environment, and genetics [2,3]. There is a field in medicine called precision medicine (or personalized medicine) that seeks to consider an individual’s characteristics to improve the prevention, diagnosis, and treatment of diseases [4].

Pharmacogenetics is considered one of the fields of personalized medicine that seeks to provide more effective treatment according to the genetic characteristics of each individual, reducing adverse effects and therapeutic failure [5]. The processes of absorption, distribution, metabolism, and excretion are crucial for its bioavailability [6].

The process of metabolizing a drug involves a series of enzymatic reactions that can be divided into phase 1 (oxidation/reduction) and phase 2 reactions (conjugation/hydrolysis) [7]. Cytochrome P450 enzymes (CYPs) are a family of hemoproteins responsible for metabolizing both xenobiotic and endogenous compounds [8]. The genes that encode these enzymes are polymorphic, as they have several variants, which means that this may account for interindividual variability in drug metabolism [9].

Genetic variants can lead to different effects on drug response: the first scenario to be observed would be during the administration of a prodrug, a pharmacologically inactive substance that requires bioactivation by metabolizing enzymes; genetic variants that result in the loss of function of these enzymes result in their non-activation and therefore do not produce the desired effect (therapeutic failure). Another scenario would be during the administration of an active drug that undergoes inactivation by the metabolizing system; in this case, loss-of-function variants may result in decreased inactivation, making drug concentrations higher and potentially generating adverse events [10].

Thus, depending on the genetic variants present in an individual, in general, four predicted phenotypes may be possible: poor metabolizers (PMs), individuals who have null enzymatic activity; intermediate metabolizers (IMs), who have reduced enzymatic activity; normal metabolizers (NMs), who have enzymatic activity within the expected rate, constituting a large part of the population; and finally, ultra-rapid metabolizers (UMs), who have increased enzymatic activity, metabolizing drugs faster than expected [9,11].

**Figure 1:**
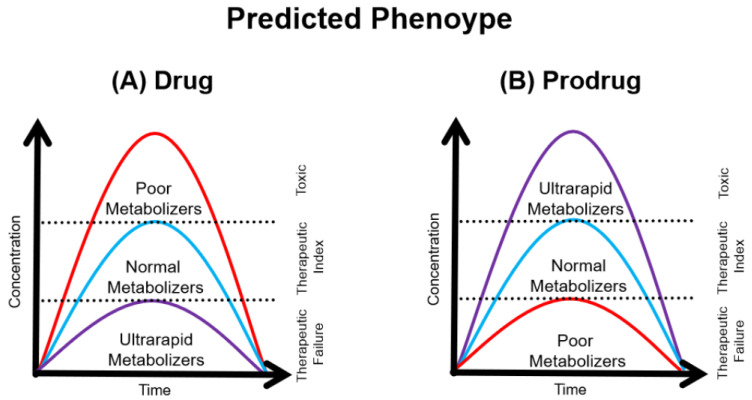
Representation of the concentration x time according to the predicted phenotypes: PMs (red), NMs (blue), and UMs (purple) when a drug (**A**) and a prodrug (**B**) are administered. The prodrug (**B**) figure represents the active metabolite concentration.

When an individual undergoes treatment with a drug, the target concentrations for the therapeutic effect are present before the metabolism that occurs to inactivate this substance. Thus, when an individual is a PM, there is a high probability of presenting adverse reactions since the drug will not be inactivated satisfactorily, so its plasma concentration will exceed the therapeutic range (Figure 1A). On the other hand, if an individual is a UM, the metabolism (and consequently inactivation) will be so fast that it will not reach the therapeutic range, and therefore, therapeutic failure is likely to occur. If the individual is treated with a prodrug, the target concentrations are those of the active metabolite, i.e., after the metabolism. PM individuals tend to have therapeutic failure because they do not generate sufficient concentrations of the active metabolite, while UM individuals may suffer adverse reactions because they produce increased concentrations of the active metabolites (Figure 1B). Therefore, through genetic testing, the predicted phenotype of an individual can be verified by predicting their metabolism rate, and in this way, the treatment can be personalized according to their peculiarities [9].

Based on predicted phenotypes, there are consortia, such as the Clinical Implementation Consortium for Pharmacogenetics (CPIC), that publish therapeutic recommendation guidelines for each phenotype group based on the evidence available in the literature. For neglected diseases, CPIC guidelines are available for HIV infection (abacavir, atazanavir, and efavirenz) and malaria (G6PD deficiency). For other drugs and diseases, such as tuberculosis, there is evidence, but no recommendation guidelines have been published yet.

In this context, this review aims to emphasize which pharmacogenetic tests are ready to be implemented in treating neglected diseases and call attention to what is missing for implementing pharmacogenetics in the treatment of HIV infection, malaria, and tuberculosis.

## 2. HIV

Human Immunodeficiency Virus (HIV) causes Acquired Immunodeficiency Syndrome (AIDS), a neglected disease that affects public health due to its complexity [12].

In 2020, 1.5 million people were diagnosed with HIV. Of this total, 65% were male homosexuals, prisoners, sex workers, transgender people, and injecting drug users. According to UNAIDS, in 2000, 24.2 million people had HIV in the world. In 2005, this number rose to 27.4 million people; in 2010, to 30.4 million; and in 2020, it reached 37.6 million people with the virus [13]. In 2022, 680,000 deaths were reported due to HIV infection [13].

HIV belongs to the Retroviridae family, presenting two single strands of ribonucleic acid—RNA [14]. It has an immunological window of approximately 30 days, which occurs from the moment of exposure until the detection of anti-HIV antibodies. It is transmitted through unsterilized sharp instruments; anal, oral, and/or vaginal sexual contact without protection; contaminated blood transfusion; and/or vertical blood transfusion (from an infected mother to her child) [15].

The first phase of AIDS is known as acute infection, when the HIV incubates from the moment of exposure until the first signs and symptoms appear, which varies between three and six weeks; the symptoms may go unnoticed because they resemble the flu, leading to lymph node enlargement, pyrexia, and torpor [16,17].

Infection prevention consists of the use of condoms, educational and awareness campaigns on Sexually Transmitted Infections, Pre-Exposure Prophylaxis (PrEP) [18], and prenatal care [19]. It is diagnosed by collecting oral fluid or blood for an anti-HIV test, or rapid tests [20].

HIV treatment emerged in 1980, consisting of antiretroviral drugs (ARVs). This class of drugs is divided into groups according to their mechanism of action: (i) reverse transcriptase inhibitors (RTIs), subdivided into nucleoside RTIs, which bind to viral DNA during reverse transcription, prematurely terminating the DNA chain (e.g., abacavir, tenofovir and lamivudine) and non-nucleoside RTIs, which bind to reverse transcriptase and block DNA synthesis (e.g., efavirenz and nevirapine); (ii) Protease Inhibitors (PIs), which bind to viral protease, which is the enzyme responsible for the maturation of immature viral proteins (e.g., ritonavir, lopinavir, atazanavir); (iii) Integrase Inhibitors, which bind to integrase, which is the enzyme responsible for funneling viral DNA to the host DNA (e.g., raltegravir, elvitegravir); (iv) Fusion Inhibitors, which prevent the viral envelope from integrating into the host cell membrane, interrupting the entry of the virus (e.g., enfuvirtide); and (v) CCR5 Inhibitors, which prevent the virus from using this channel to enter T lymphocytes (e.g., maraviroc) [20].

The metabolism of these drugs occurs mainly in the liver in enzymes of the cytochrome P450 complex, and in phase II enzymes, and their main adverse effects include gastrointestinal, hematological, hepatic toxicity, and impacts on the Central Nervous System [9].

In the context of the pharmacogenetics of the treatment for HIV infection, CPIC contains guidelines for treatment with abacavir, atazanavir, and efavirenz [21]

Abacavir is a nucleoside RTI that can cause hypersensitivity reactions in some individuals. Such reactions can be serious and even lead to death, so the identification of patients at risk of hypersensitivity reactions is necessary. According to previous studies and the CPIC guideline [22], the genotyping of the HLA-B*57:01 variant in the *HLA-B* gene is useful for identifying patients most likely to present hypersensitivity reactions, which consist of dyspnea, rash, fatigue, pyrexia, gastrointestinal symptoms, and cough. If the patient presents two or more symptoms, the use of the medication should be discontinued, and an alternative therapy should be introduced since the continuation of this medication in these cases can lead to death [23].

The presence of HLA-B*57:01 is associated with diseases such as inflammatory bowel disease, ankylosing spondylitis, hepatotoxicity, skin lesions, psoriasis, Stevens–Johnson syndrome, and uveitis. In general, approximately 94% of patients have a low risk of hypersensitivity (absent HLA-B*57:01—negative), and 6% of patients have a high risk of hypersensitivity because they have at least one HLA-B*57:01 allele (positive). According to the CPIC guideline, in cases when the patient tests positive for the HLA-B*57:01 allele, it is recommended not to prescribe abacavir and use an alternative treatment (Table 1). The frequency of this allele is low or practically absent in some African and Japanese populations, while in Europeans, this frequency can reach up 7%, and in Southwest Asians, up to 20% [23].

Atazanavir is an antiretroviral drug metabolized by the uridine diphosphate glucuronosyltransferase—the UGT1A1 enzyme encoded by the *UGT1A1* gene. This enzyme is expressed in the liver and gastrointestinal tract, helping in the elimination of bilirubin and the catabolism of heme. Therefore, its reduced activity can cause jaundice and Gilbert’s syndrome, and increased bilirubin in the blood can trigger neurological adverse effects. The *UGT1A1* gene presents variants of normal function (*1), increased function (*36), or reduced function (*6, *28, and *37). Normal metabolizers consist of individuals with the reference allele (*1), or with increased function alleles (*36) and homozygous rs887829C/C, and are not recommended to change treatment with atazanavir. Intermediate metabolizers have the reference allele (*1) or increased function allele (*36) together with decreased function alleles (*6, *28, *37) and/or heterozygous rs887829 C/T, with a low probability of bilirubin-related discontinuation of atazanavir, and poor metabolizers include those with two decreased function alleles and/or homozygous for rs887829 T/T (*80/*80), with a recommendation to consider an alternative therapy due to the high risk of adverse events (Table 1). According to the CPIC, the classification of these recommendations is strong [24].

Efavirenz is a non-nucleoside RTI, and is metabolized by the CYP2B6 enzyme, of which its gene variants can lead to nervous system toxicity. Ultra-rapid metabolizers have two alleles of increased function (*4, *22), rapid metabolizers have one allele of normal function (*1) and one allele of increased function, and normal metabolizers have two alleles of normal function. According to the CPIC guideline, all these three predicted phenotypes are recommended to start the drug with a standard dose of 600 mg/day (Table 1—strong recommendation) [25]. Intermediate metabolizers have one allele of normal function and one allele of decreased function (*6, *9), or one allele of normal function and one allele of null function (*18), or one allele of increased function and one allele of decreased function, or one allele of increased function and one allele of null function, and it is recommended to start the medication with a reduced dosage of 400 mg/day due to the risk of adverse events of the Central Nervous System. Poor metabolizers have two alleles of decreased function or two alleles without function or one allele of decreased function and one allele of null function, and it is recommended to start the medication with a reduced dosage of 400 or 200 mg/day due to the high risk of adverse events of the Central Nervous System (Table 1). According to the CPIC, the classification of the IM and PM recommendations are moderate [25].

## 3. Malaria

Malaria is a hemoparasitosis with a major impact on public health, transmitted through the bite of female mosquitoes of the genus *Anopheles* sp., infected by the protozoa of the genus *Plasmodium* sp. To date, seven species have been described as having infected humans, namely *P. falciparum*, *P. vivax*, *P. ovale*, *P. malariae*, *P. knowlesi*, *P. cynomolgi*, and *P. simium* [26,27,28].

In 2022, an estimated 249 million cases worldwide were recorded in 85 malaria-endemic countries, an increase of 5 million compared to 2021. The African region accounted for 94% of cases globally, and the South-East Asia Region accounted for 2%. According to the WHO, the Eastern Mediterranean Region between 2015 and 2022 presented a rise in cases of 92% to 8.3 million, whereas in the Americas, between 2000 and 2022 malaria cases declined by 64.0% (from 1.5 million to 0.55 million), and the incidence declined by 72.5% (from 13.1 to 3.6 cases per 1000 population at risk). Venezuela, Brazil, and Colombia were the countries responsible for the highest percentage of cases (73%) [29].

The biological cycle begins during the blood meal, when the female mosquito inoculates sporozoites (the infective form for the vertebrate host) into the host’s skin; it is through this process that the interaction of proteins from the parasite’s apical complex with the epidermis occurs, and the invasion process begins; the parasite migrates toward the hepatocytes, initiating the extra-erythrocytic (or hepatic) cycle; once the hepatocyte is invaded, the first asexual reproduction occurs, resulting in merozoites, which are released into the peripheral circulation within merosomes that are later ruptured [30].

In some species such as *P. vivax* and *P. ovale*, the parasites can remain dormant for long periods (hypnozoites) and can be reactivated, leading the host to relapse [31]. Merozoites invade red blood cells, initiating erythrocytic schizogony, where the parasite develops into an immature trophozoite stage (ring form), a mature trophozoite stage, and a schizont stage, which will give rise to new merozoites that will be released into the bloodstream, where they will consequently invade other red blood cells. This asexual replication can also give rise to the sexual stages of the parasite (gametocytes), which can be ingested by an uninfected female mosquito during a blood meal, perpetuating the cycle [31].

The main objectives of malaria treatment are to (1) interrupt blood schizogony; (2) destroy latent forms (hypnozoites) to prevent relapses; and (3) interrupt the transmission of the parasite to the vector using drugs that prevent the development of sexual forms (gametocytes). The main antimalarial drugs can be divided by their target of action in the cycle: tissue schizonticides or hypnozoiticides, blood schizonticides, gametocytocides, and sporonticides. For *P. vivax* or *P. ovale*, the main objective of treatment is to combat both blood and latent forms, preventing cases of recrudescence and relapse, respectively [32].

Dosages for treating malaria will depend on the individual’s age and weight and may also vary according to the parasite species [32]. In Brazil, for uncomplicated malaria caused by *P. vivax* or *P. ovale*, the treatment regimen consists of Chloroquine (CQ) for three days, together with primaquine (PQ) for seven days, except for pregnant women, lactating women during the first month, and children up to 6 months, in whom primaquine is contraindicated [32]. Recently, tafenoquine (TAQ), an analog of PQ with a longer half-life and in a single dose, was approved for use in treatment, but it is still in the implementation phase [32,33].

The treatment for *P. malariae* is quite similar to that for vivax malaria, but without the need for PQ. For falciparum malaria, the World Health Organization (WHO) recommends the use of ‘artemisinin-based combination therapies’ (ACTs) [29]; the use of Artemether/Lumefantrine or Artesunate/Mefloquine is recommended, depending on availability; PQ should be administered in a single dose on the first day of treatment at a low dose to ensure the elimination of circulating mature gametocytes [29,32].

For carriers of G6PD deficiency, a genetic condition linked to the X chromosome that can remain asymptomatic for the rest of life unless the individual is exposed to certain conditions, it is recommended that testing be carried out before starting treatment if it is available in the health service, due to the risk of hemolysis that can be induced by PQ/TAQ [29,32]. Individuals with G6PD deficiency with <20% activity or between 20 and 45% activity may be at increased risk of hemolysis when treated with the standard PQ regimen [32,34]. Some studies suggest that CYP2D6 IMs or PMs may respond poorly to the radical cure treatment of vivax malaria, resulting in recurrences [35]. Also, the CPIC *G6PD* guideline presents a strong recommendation to avoid or change the dosage of PQ treatment in individuals with G6PD deficiency, considering their high risk of hemolytic anemia [36].

Considering the metabolism of antimalarial drugs by CYPs, a study was conducted in Zanzibar to assess the influence of *CYP2C8* polymorphisms in patients who had been treated with artemether-amodiaquine (AS-AQ) for uncomplicated *Plasmodium falciparum* malaria. The frequency of CYP2C8*2 was 17.5% (95% CI 15.4–19.7) and that of CYP2C8*3 was 2.7% (95% CI 1.8–3.7), but there was no significant difference between those with re-infections or those recrudescent groups (4.1%; 95% CI: 33.8–54.8 vs. 8.3%; 95% CI: 29.4–67.5) when compared to those with and adequate clinical and parasitological response (36.7%; 95% CI: 30.043.9) (*p* = 0.25 and *p* = 0.31, respectively). Interestingly, the incidence of adverse events after treatment with AS–AQ was higher in subjects carrying either the CYP2C8*2 or CYP2C8*3 alleles (44.9%; 95% CI: 36.1–54.0), suggesting that *CYP2C8* genotypes did not appear to influence treatment efficacy, but the tolerability may be reduced in subjects carrying CYP2C8 *2, *3 alleles [37].

Pett et al., 2019 [35], aimed to determinate the impact of the genetically inferred CYP2D6 metabolizer status on the gametocytocidal and hemolytic effect of single-dose PQ in eight clinical trials conducted across Africa, and observed in patients who had gametocytes that the prevalence of CYP2D6 PM/IM status was 31.4% (171/544) overall, and those individuals had a higher gametocyte prevalence on day 7 or 10 after PQ than those with an extensive/ultrarapid CYP2D6 metabolizer status (odds ratio [OR]: 1.79 [95% confidence interval {CI}: 1.10, 2.90]; *p* = 0.018), suggesting that it may be associated with prolonged gametocyte carriage after treatment with single-low-dose PQ.

The pregnancy and pharmacogenetic variation were observed in patients treated with artemether-lumefantrine (AL) in Tanzania [38]. The mean of 7 days was significantly lower in pregnant women than non-pregnant women [geometric mean ratio = 1.40; 95% confidence interval (CI) of geometric mean ratio (1.119–1.1745), *p* < 0.003]. Besides that, variables like pregnancy, low body weight, and CYP3A5*1/*1 genotype were significantly associated with low day 7 LF plasma concentration (*p* < 0.01), suggesting significant predictors of low day 7 lumefantrine plasma exposure, which is associated with a higher risk of recrudescence.

The potential pharmacogenetic variations in response to generic AL in the treatment of uncomplicated *P. falciparum* malaria was evaluated in Ghana [39]. On day 3, a significant difference in plasma dihydroartemisinic concentrations, an active metabolite, was noted among the various CYP2B6*6 genotypes (*p* = 0.0031), and there was a notable difference in plasma desbutyl lumefantrine concentrations, another active metabolite (*p* = 0.0019), between CYP3A5*6 carriers, showing that *CYP2B6* and *CYP3A5* pharmacogenetic variations may lead to a higher plasma exposure of AL metabolites in these patients.

In Korea, Choi et al. observed in patients diagnosed with vivax malaria a total of 16 *CYP2D6* alleles: the most common genotype was *10B/*10B, phenotypes PM+IM were more common in the recurrence group with an OR of 2.33 (95% CI, 1.14–4.77, *p* = 0.02), and when the association between recurrence and the activity scores was analyzed, patients with higher activity scores were significantly less likely to have a recurrence (*p* = 0.028), which suggests that *CYP2D6* polymorphism may affect primaquine efficacy and thus *Plasmodium vivax* recurrence in this country [40].

Dowd et al., 2023 [41], investigated the relationship between the CYP2D6 activity status and tafenoquine (TQ) efficacy in preventing *P. vivax* relapses in Australian Defense Force personnel deployed to Papua New Guinea and Timor-Leste; the authors observed a higher frequency of the wild-type genotype *1/*1 (29.3%) followed by *1/*4 (9.8%) and *4/*4 (7.6%), but it was not significant between the non-relapse and relapse groups (*p* > 0.05). Curiously, the risk of relapse was ever higher for individuals predicted to be IM, with a homologous allele combination (OR = 13.13; 95% CI: 1.92–89.52) but no significant difference was observed in the proportions of CYPD6 NMs, IMs, and PMs between the groups, indicating that the CYP2D6 activity status did not affect the relapse outcome between patients treated with TQ.

Spring et al. aimed to determine the *CYP2D6* genotype and predicted the phenotype in a cohort of US military personnel. In total, 76 different *CYP2D6* genotypes were observed (n = 530), with the following four genotypes having a frequency of ≥5%: *1/*2 (13.2%), *1/*1 (12.8%), *1/*4 (8.5%), and *1/*41 (5%). When the plasma PQ PK profile was evaluated according to the activity score, those with AS-As of 2, 1.5, and 1 had similar profiles, while the five volunteers in the IM group with an AS-A of 0.25 (*10/null allele) had a PK profile that was almost identical to that for the PM group, suggesting that PQ metabolism is decreased in individuals with the IM or PM phenotypes compared to those with the NM phenotype [42].

In Brazil, the most prevalent variants observed were *CYP2D6* C100T and G1846A and CYP2C8*3 (G416A). For the *CYP2D6* C100T variant, a significantly higher frequency of heterozygous and homozygous mutant genotypes was observed in individuals who presented multiple relapse infections (12/18 [66.7%] vs. 7/28 [25.0%]; *p* = 0.007). For the CYP2C8*3 (G416A) allele, it was observed that a higher proportion of mutated individuals had a recurrence of *P. vivax* malaria; therefore, the chances of the disease returning in these individuals increased 4.8 times in patients who presented this variant [43].

Another Brazilian study showed that individuals with the wild-type genotype, when compared to carriers of the slow-metabolizing alleles of CYP2C8 *2, *3, and *4, achieved a greater reduction in their gametocyte levels (*p* = 0.007) than individuals without this genotype [44].

In a prospective cohort study conducted in the Brazilian Amazon, it was observed that CYP2D6 PMs or IMs presented an increased risk of *P. vivax* recurrences after CQ + PQ therapy when compared to NMs or UMs, due to a possible reduction in the conversion of PQ into its active metabolites, thus decreasing its hypnozoiticidal potential [45].

Aiming to investigate the influence of CYP genotypes associated with CQ metabolism on early recurrence rates of *P. vivax*, a study in the Brazilian Amazon observed similar frequencies of CYP2C8*2, *3, and *4 (*p* = 0.3196), CYP3A4*1A and *1B (*p* = 0.0916), and CYP3A5*3 and *6 (*p* = 0.1064) alleles related to normal and poor metabolism between the recurrent and non-recurrent groups, in addition to the levels of CQ and its metabolite desethylchloroquine (DCQ) also being very similar between the groups despite the presence of the observed variants, with no significant differences [46].

Cardoso et al., 2022 [47], sought to investigate possible associations between *CYP2D6, CYP2C19*, and *CYP3A4* variants and predicted phenotypes among vivax malaria recurrences. The frequencies found were similar between the observed groups for CYP2C19*2 (9.8% vs. 10.5%, *p* = 0.924), CYP3A4*1B (*p* = 0.841), and *CYP2D6* null function alleles (*4 and *5), decreased function (*9, *10, *17, *29, and *41), and normal function alleles (*1, *2, *35, and *39) with *p* > 0.05. Only the CYP2D6*2xN allele (*p* = 0.047) and the *CYP2D6* gUM phenotype (*p* = 0.057) were more frequent in individuals in the non-recurrent group. Despite these findings, none of the variants studied were associated with an increased risk of recurrence, nor did they show any influence on parasite elimination or the time to the number of recurrence episodes.

Puça et al., 2024 [48], investigated the influence of monoamine oxidase-A (MAO-A) on PQ metabolism and its impact on vivax malaria relapses; the authors observed that 30% (*n* = 100) impaired CYP2D6 activity, inferred from genotype data (AS ≤ 1.0), and 31% carried low-expression MAO-A variants; however, no differences in PQ levels were documented between high- and low-expression MAO-A variants [median = 179.0 ng/mL (IQR = 146.0, 232.0) and 188.0 ng/mL (119.0, 217.5), respectively, *p* = 0.907] in impaired CYP2D6 activity. Besides that, the median carboxyprimaquine level was significantly reduced in the specific group of subjects carrying low-expression/activity alleles in both *MAO-A* and *CYP2D6* (*p* = 0.034) showing a potential gene–gene interaction where the status of the *MAO-A* promoter region is critical, supporting the hypothesis that this metabolite may be further metabolized through CYP-mediated pathways generating bioactive metabolites that act against the parasite.

Silvino et al., 2020 [49], evaluated how genetic variants in *CYP2D6* could influence the outcome of malaria treatment with PQ in a population in the Brazilian Amazon; the most common diplotypes observed were *2/*4 with 7.3%, *1/*4 with 6.9%, *1/*5 with 4.2%, and *17/*17 with 1.5%. In summary, the frequency of phenotypes with reduced CYP2D6 activity (AS ≤ 1.0) was higher in individuals with recurrence (32.4% and 27.5% for the multiple- and single-recurrence groups, respectively, versus 20.8% for the no-recurrence group; χ^2^  =  3.402, *p*  =  0.065).

Macedo et al., 2023 [50], investigated the association between variants of *CYP2C19, CYP2D6*, and *CYP3A4* with hemolysis by PQ in patients with G6PD deficiency; no significant differences were observed in the frequency of star alleles between the deficient and non-deficient groups (*p* > 0.05); however, the authors observed that G6PDd patients had the worst hemolytic condition during hospitalization, and when comparing the predicted phenotypes of *CYP2C19* and *CYP2D6* with the clinical and laboratory profile, it was possible to identify that individuals with gRM and gUM had the highest serum levels of hemolysis markers. In addition, the CYP3A4 *1/*1B genotype had the greatest clinical impact in both G6PD groups, but with no significant differences. The findings reinforce the importance of studies in a larger population with G6PD deficiency to better understand which possible associations can be identified between the genetic variants of *CYP2C19, CYP2D6,* and *CYP3A4* and the hemolytic process during vivax malaria infection.

Despite the mentioned CYP evidence, no clinical pharmacogenetic recommendation considering these genes’ variants was published for the treatment with antimalarial drugs yet.

## 4. Tuberculosis

In 2022, 7.5 million people were diagnosed with tuberculosis worldwide, with 5.8 million cases in men (55%), and 1.3 million cases in children aged 0 to 14 (12%). In 2022, tuberculosis was considered the second leading cause of death from an infectious disease, with approximately 1.30 million deaths worldwide. Globally, it was found that Southwest Asia accounts for 46%, Africa for 23%, the Western Pacific for 18%, the Eastern Mediterranean for 8.1%, the Americas for 3.1%, and Europe for 2.2% of the total number of infected people in the world [51].

Tuberculosis is caused by *Mycobacterium africanum*, *M. bovis*, *M. microti*, and/or *M. tuberculosis*. Its main form of contagion is through the inhalation of contaminated aerosolized particles. It can be divided into three stages that vary according to virulence and risk groups: primary infection, latent infection, and active infection [52].

In primary infection, which is usually asymptomatic, aspirated bacilli settle in the upper airways and lungs, mainly in the alveoli, middle and/or lower lobes, activating macrophages to perform phagocytosis. However, mycobacteria not destroyed by macrophages inhibit the junction of the phagosome with the lysosome and multiply. In the immune system, the innate immune response is triggered, releasing cytokines such as TNF-α and IL-12 [52].

In latent infection, bacilli located in the lungs and other locations change into granulomas of epithelioid cells with a caseous center, as soon as the adaptive immune response is activated, releasing TCD4+ lymphocytes and TCD8+ lymphocytes, preventing the spread of the bacillus. In cases of risk factors, Simon’s foci may appear, that is, hematogenous dissemination and/or Ghon’s foci, which are consolidations in the lungs and extrapulmonary lymphadenopathy [52].

From the onset of signs and symptoms that include anorexia, cachexia, fatigue, pyrexia, and productive cough, active infection occurs, with tissue damage due to delayed-type hypersensitivity with granulomatous necrosis. Complications include pleural effusion, dyspnea, empyema, bronchopleural fistula, hemoptysis, and pneumothorax, among others [52].

Diagnosis is made through imaging tests, such as radiography, culture and acid-fast staining [53], interferon **γ** release assay, tuberculin skin tests, and/or nucleic acid amplification tests [54].

Tuberculosis treatment consists of anti-tuberculosis drugs (such as isoniazid, rifampicin, pyrazinamide, and ethambutol). The individual responses for these drugs were associated with genes related to their metabolism and transport, such as *NAT2*, *ABCC2*, *GSTP1*, *NOS2*, and *CYP2C9*.

A recent review about this subject identified several key findings regarding the influence of genetic variants on the response to tuberculosis treatment [55]. Variants in the *NAT2* gene, which plays a crucial role in the metabolism of isoniazid, were associated with varying levels of risk or protection against hepatotoxicity. For instance, the *4 allele was observed to have a protective effect in several populations, whereas the *6 and *7 alleles were linked to an increased risk of adverse reactions. These findings highlighted significant ethnic differences, with Asian populations being particularly affected.

Another important discovery involved the *ABCC2* gene, where the rs3740065 variant emerged as a protective factor against drug-induced hepatotoxicity. Similarly, the *GSTP1* gene was associated with a higher risk of liver injury in individuals carrying specific variants, such as rs1695.

Additional genes, including *CYP2C19*, *CYP2C9*, and *NOS2*, were also significantly associated with treatment outcomes and adverse effects, further underscoring the complex interplay between genetic factors and drug response in tuberculosis therapy. These findings emphasize the potential of genetic profiling to improve treatment safety and efficacy across diverse populations.

Recently Shabani et al., 2024 [56], investigated the influence of pharmacogenetics markers in the *NAT2* and *SLCO1B1* genes on tuberculosis treatment in the Iranian population; the *NAT2* gene is responsible for isoniazid metabolism, and *SLCO1B1* is responsible for rifampicin separately. For the *NAT2* gene, seven variants in exon 2 were found: rs1041983, rs1801280, rs1799929, rs1799930, rs1208, rs1799931, and rs2552. These all cause reduced enzyme activity, and consequently, a slow acetylation phenotype, which may lead to liver toxicity. For the *SLCO1B1* gene, a frequency of 12.9% for rs4149056 was found and the presence of this variant might cause a decrease in *SLCO1B1* expression, resulting in the reduced uptake of OATP1B1, which leads to high levels of rifampicin in the bloodstream.

In Ethiopian patients, the variability in plasma rifampicin concentrations and the role of the *SLCO1B1*, *ABCB1*, arylacetamide deacetylase (*AADAC*), and carboxylesterase 2 (*CES-2*) genotypes was not explained by SLCO1B1*1B (c.388A>G) and SLCO1B1 *5 c.521T>C (associated with the increased and decreased transporter activity of OATP1B1, respectively). Also, there was no significant influence of *ABCB1* c.3435C>T and *CES-2* c.269-965A>G on rifampicin plasma concentrations. However, rifampicin exposure varied with sex, dose and *ABCB1* c.4036A>G and *AADAC* c.841G>A genotypes. Male patients with *AADAC* c.841GG and *ABCB1* c.4036AA genotypes showed a significant higher risk of low rifampicin plasma exposure than females [57].

Jaramillo-Valverde et al., 2022 [58] studied the possible association with the development of drug-induced liver injury and *NAT2* and *CYP2E1* polymorphisms in Peruvian patients; those findings showed that patients who had NAT2*4/*5 and had the *CYP2E1 c1/c1* genotype had significant protection (OR: 0.16; 95% CI: 0.00–1.25; *p* = 0.049) against developing drug-induced liver injury compared with the most prevalent combination between the *NAT2* and *CYP2E1* genotypes (NAT2*5/*5 and *CYP2E1 c1*/*c1*). 

In Latvia, Ulanova et al., 2024 [59], investigated the relationships between isoniazid exposure and isoniazid metabolism-related genetic factors in the occurrence of drug induced hepatotoxicity and tuberculosis treatment. During the univariate analysis, there was no significant association between treatment outcome and genetic factors; however, the drug induced hepatotoxicity occurred only in patients with *NAT2* slow activity phenotype (*N =* 4). Three isoniazid pharmacokinetic parameters [increase in INH AUC0–6h, and decrease in both (acetylisoniazid) AcINH/INH and (isonicotinic acid) INA/INH ratios] between CYP2E1*6 (rs6413432) showed statistical significance (*p* < 0.05), indicating that *CYP2E1* significantly affects the isoniazid pharmacokinetics along with *NAT2*.

The relationship between genetic variants of *NAT2* and *CYP2E1* with the plasma concentration of isoniazid and acetylisoniazid and their possible association with hepatotoxicity was observed in Iranian patients with tuberculosis [60]. The genetic analysis indicated that the 40 patients with hepatoxicity were genotyped as intermediate activity and slow activity for the *NAT2* gene (20–80% respectively). The risk of anti-tuberculosis drug-induced hepatotoxicity was significantly higher in slow-activity patients (*p* < 0.001). The frequency of NAT2*5 (*p* = 0.003) and NAT2*6 (*p* < 0.001) haplotypes was significantly higher in patients with hepatotoxicity, and *NAT2* slow activity with *CYP2E1 c1/c1* genotype in patients with hepatotoxicity was significantly different from patients without hepatoxicity (*p* < 0.001), showing a higher prevalence of hepatotoxicity in this group of the Iranian population.

Haas et al., 2022 [61], investigated associations between genetic polymorphisms and individual variability in plasma clearance of bedaquiline, an oral agent used in cases of resistance to first- and second-line anti-tuberculosis drugs, its M2 metabolite, and clofazimine in South Africa. The variant CYP3A5*3 (rs776746) was associated with slower clearance of bedaquiline (*p* = 0.0017), and a genome-wide significant association between *CNTN5* rs75285763 and slower plasma clofazimine clearance (*p* = 2.9 × 10^−8^) was also found.

## 5. General Considerations

While pharmacogenetic testing for HIV infection, malaria, and tuberculosis shows great promise, there are some issues to be resolved. For HIV infection, the availability of validated tests represents a milestone, but their wider adoption in resource-limited settings remains a critical challenge. For malaria and tuberculosis, pharmacogenetic testing is not yet fully validated, requiring significant investment in research.

The limited availability of effective drugs to treat infectious diseases, coupled with the growing therapeutic failure of existing treatments, underscores the urgent need to identify actionable therapeutic targets in the causative organisms. Precise drug administration with a personalized dosage enhances the likelihood of eradicating the infectious agent and consequently contributes significantly to the control of these diseases. Furthermore, it is important to use pharmacogenetics in the treatment of infectious diseases to enhance treatment efficacy, minimize drug resistance, reduce adverse drug reactions, guide new drug development, and improve the cost-effectiveness of the treatments.

There are some studies comprising the cost-effectiveness of treatments using pharmacogenetic tests, but they are often conducted in developed countries and involve the treatment of diseases such as cancer and cardiovascular issues. A systematic review presented some data about the cost-effectiveness of pharmacogenetic tests in developing countries, and most studies reported the testing to be cost-effective [62]. However, the authors discuss that besides the costs, some challenges to implement PGx in low- and middle-income countries are the lack of clinical trials to validate the biomarkers of interest, low resources, cultural issues, and low interest of investors. One of the included studies comprised a tuberculosis and *NAT2* test for isoniazid treatment in Brazil, India, and South Africa and concluded that it is cost-effective in all these three countries [63]. Another systematic review included studies that analyzed the cost-effectiveness of abacavir, efavirenz, and atazanavir tests in Spain, Singapore, Germany, Swiss, USA, and the UK, and found that most results were cost-saving or cost-effective [64].

Moreover, one of the greatest problems related to the treatment of the neglected diseases is the lack of adherence to treatment caused by the lack of access to healthcare systems or medications themselves, or also due to the adverse effects that these treatments can cause, resulting in treatment abandonment. Pharmacogenetic tests, at first, can add complexity to the implementation; however, in hospitals or health centers with a minimum of structure (a basic molecular biology laboratory with real-time PCR equipment would be necessary), it may be possible carrying out such an implementation, personalizing the treatment, in addition to increasing cure rates, reducing therapeutic failures and adverse effects, and contribute to reducing treatment abandonment.

## 6. Conclusions

Pharmacogenetics is very useful for predicting drug response in a variety of diseases. In the context of neglected diseases, there are two major barriers: (i) the lack of evidence due to the few studies on the diseases themselves and (ii) the admixture of the most affected populations, which must be taken into account, given the genetic differentiation of these populations.

Some tests are already being implemented, such as the abacavir, atazanavir, and efavirenz tests. However, with this study, we would like to draw attention to the need to generate more evidence for testing treatments for other neglected diseases, such as malaria and tuberculosis, given their epidemiological importance and for the public health of less favored populations.

The current landscape of pharmacogenetics for neglected diseases shows both promise and disparity. It will be important to bridge the gap between researchers, policymakers, and health organizations to ensure that pharmacogenetics fulfills its potential to improve treatment outcomes for the most vulnerable populations.

## Figures and Tables

**Table 1:** Summary of CPIC therapeutic recommendations for genotype/phenotype of individuals treated with abacavir, atazanavir, and efavirenz [22,24,25].

Drug	Gene	Likely Phenotype	Genotypes/Diplotypes	Recommendations
Abacavir	*HLA-B*	Very low risk of hypersensitivity	Absence of *57:01 alleles (reported as “negative” on a genotyping test). *X/*X^a^	Use abacavir per standard dosing guidelines.
		High risk of hypersensitivity	Presence of at least one *57:01 allele (reported as “positive” on a genotyping test). *57:01/*X *57:01/*57:01	Abacavir is not recommended.
Atazanavir	*UGT1A1*	Normal metabolizer	An individual carrying two reference function (*1) and/or increased function alleles (*36). Alternatively identified by homozygosity for rs887829 C/C. *1/*1; *1/*36; *36/*36; rs887829 C/C	There is no need to avoid prescribing atazanavir based on *UGT1A1* genetic test result. Inform the patient that some patients stop atazanavir because of jaundice (yellow eyes and skin), but that this patient’s genotype makes this unlikely (less than about a 1 in 20 chance of stopping atazanavir because of jaundice).
		Intermediate metabolizer	An individual carrying one reference function (*1) or increased function allele (*36) plus one decreased function allele (*6, *28, *37). Alternatively identified by heterozygosity for rs887829 C/T. *1/*28; *1/*37; *36/*28; *36/*37; rs887829 C/T, *1/*6	There is no need to avoid prescribing atazanavir based on *UGT1A1* genetic test result. Inform the patient that some patients stop atazanavir because of jaundice (yellow eyes and skin), but that this patient’s genotype makes this unlikely (less than about a 1 in 20 chance of stopping atazanavir because of jaundice).
		Poor metabolizer	An individual carrying two decreased function alleles (*6, *28, *37). Alternatively identified by homozygosity for rs887829 T/T (*80/*80). *28/*28; *28/*37; *37/*37; rs887829 T/T (*80/*80), *6/*6	Consider an alternative agent, particularly where jaundice would be of concern to the patient. If atazanavir is to be prescribed, there is a high likelihood of developing jaundice that will result in atazanavir discontinuation (at least 20% and as high as 60%).
Efavirenz	*CYP2B6*	Ultrarapid metabolizer	An individual carrying two increased function alleles. *4/*4, *22/*22, *4/*22	Initiate efavirenz with standard dosing (600 mg/day).
		Rapid metabolizer	An individual carrying one normal function allele and one increased function allele. *1/*4, *1/*22	Initiate efavirenz with standard dosing (600 mg/day).
		Normal metabolizer	An individual carrying two normal function alleles. *1/*1	Initiate efavirenz with standard dosing (600 mg/day).
		Intermediate metabolizer	An individual carrying one normal function allele and one decreased function allele OR one normal function allele and one no-function allele OR one increased function allele and one decreased function allele OR one increased function allele and one no-function allele. *1/*6, *1/*18, *4/*6, *4/*18, *6/*22, *18/*22	Consider initiating efavirenz with decreased dose of 400 mg/day.
		Poor metabolizer	An individual carrying two decreased function alleles OR two no-function alleles OR one decreased function allele and one no-function allele. *6/*6, *18/*18, *6/*18	Consider initiating efavirenz with decreased dose of 400 or 200 mg/day.

^a^ *X—any HLA-B genotype other than *57:01.

## Data Availability

Data sharing is not applicable.
